# Rhizobial gibberellin negatively regulates host nodule number

**DOI:** 10.1038/srep27998

**Published:** 2016-06-16

**Authors:** Yohei Tatsukami, Mitsuyoshi Ueda

**Affiliations:** 1 Division of Applied Life Sciences, Graduate School of Agriculture, Kyoto University, Sakyo-ku, Kyoto 606-8502, Japan; 2 Japan Society for the Promotion of Science, Sakyo-ku, Kyoto, Japan

## Abstract

In legume–rhizobia symbiosis, the nodule number is controlled to ensure optimal growth of the host. In *Lotus japonicus*, the nodule number has been considered to be tightly regulated by host-derived phytohormones and glycopeptides. However, we have discovered a symbiont-derived phytohormonal regulation of nodule number in *Mesorhizobium loti*. In this study, we found that *M. loti* synthesized gibberellic acid (GA) under symbiosis. Hosts inoculated with a GA-synthesis-deficient *M. loti* mutant formed more nodules than those inoculated with the wild-type form at four weeks post inoculation, indicating that GA from already-incorporated rhizobia prevents new nodule formation. Interestingly, the genes for GA synthesis are only found in rhizobial species that inhabit determinate nodules. Our findings suggest that the already-incorporated rhizobia perform GA-associated negative regulation of nodule number to prevent delayed infection by other rhizobia.

Rhizobia are soil bacteria that fix nitrogen after establishing symbiosis with legume family (Fabaceae). Legume–rhizobia symbiosis is one of the best-studied mutualisms among plant–microbe interactions. Rhizobial infection of compatible legume strains leads to nodule formation through complex signal exchanges. This relationship is based on the reciprocal provision of a photosynthetic carbon source from the host and fixed nitrogen sources from the symbiont. Appropriate nodule formation is a very effective means of host survival in nitrogen-deficient soil; however, over-nodulation consumes too much energy for nitrogen fixation and nodule organogenesis. To optimize the ratio of nitrogen income to carbon-based energy loss, the host plant tightly regulates nodule organogenesis. Nodule development is locally and systemically controlled^1^,^2^. In local control, several phytohormones are induced in plant in response to rhizobial Nod-factors (NFs) such as lipopolysaccharide and exopolysaccharide secreted by rhizobia^2^. In systemic control, plants perform autoregulation of nodulation (AON), which is a long-distance root-to-shoot-to-root negative feedback system^3^,^4^,^5^. In this system, plants use CLV3/ESR-related peptides as root-to-shoot signal molecules in response to high nitrate condition and infection of rhizobia^6^,^7^, and use cytokinin as a shoot-to-root signal^8^. In both cases, nodule organogenesis is reported to be controlled by plant-derived phytohormones and peptides^2^.

Of these signal molecules, GAs are known to play a role in the control of nodule development^9^. GAs produced by higher plants usually regulate a wide range of plant growth and influence various developmental processes. GA-synthetic genes in higher plants are upregulated during nodulation^10^,^11^, and GA-deficient mutants form fewer nodules than wild-type plants^12^. Exogenous addition of GAs also affects nodulation. Low concentrations of GA_3_ are reported to increase nodule formation in *Pisum sativum*^13^, but decrease nodule number in *Lotus japonicus*^14^. These results show that an optimum level of GAs is required for proper nodulation.

While nodule number is presumed to be under control of host plant-derived signal molecules, in this study, we have found that *M. loti* synthesized GA with symbiosis-specific expressing operon, and that the GA produced by already-incorporated rhizobia have the function to reduce the host nodule number. Moreover, we found the clear relationships between distribution of GA-synthetic genes and host nodule types. Our results suggest that rhizobial GA negatively regulates the host nodule number to be beneficial to already-incorporated rhizobia. This is the first report suggesting that incorporated rhizobia negatively affect the nodule number via synthesis of phytohormone.

## Results

### Functional analysis of a putative GA operon in *M. loti*

**Figure 1:**
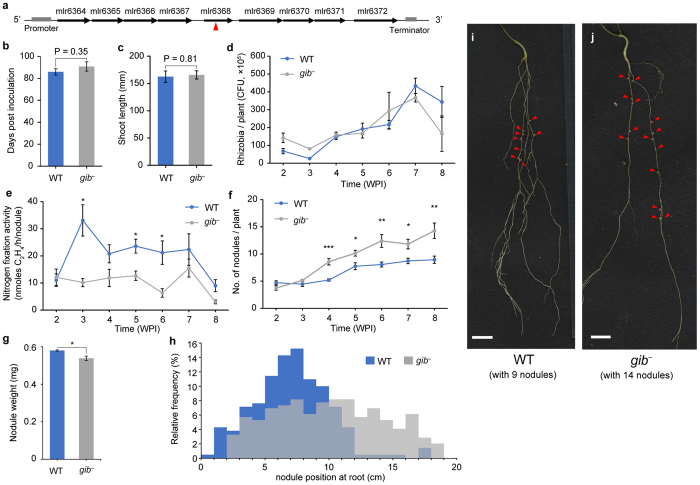
GA-synthetic operon in *M. loti* and time-course plant assays. (**a**) The operon consists of 9 genes with accession numbers mlr6364–mlr6372. The arrowhead indicates the transposon insertion site in the *gib*^*−*^ mutant. (**b**) Flowering period from the day of inoculation. (**c**) Shoot length of the host plant when flowering. (**d**) Number of rhizobia grown in roots. (**e**) Nitrogen fixation activity determined by acetylene reduction assay. (**f**) Number of nodules. (**g**) Weight per nodule at 5WPI. Bars indicate the average weights of 30 nodules. (**h**) Relative frequency of nodule position from root-stem boundary at 8WPI. Significance was determined by Mann-Whitney *U*-test (p-value = 5.52 × 10^−13^). Relative frequencies were calculated by 210 for wild-type and 245 nodules for *gib*^*−*^ mutant, respectively. (**i,j**) Nodules formed in root of *L. japonicus* inoculated with *M. loti* wild-type and *gib*^*−*^ mutant at 8WPI. Red arrows indicate the nodules. Significances were determined by student *t*-test (**b,c,g**). Multiple comparisons were corrected using the Holm–Bonferroni *t*-test. *p < 0.05, **p < 0.01, ***p < 0.001 (**d,e,f**). WPI, weeks post inoculation. Error bars indicate SEMs from 3 plants (**d**), 8 plants (**e**), >15 plants (**b,c,f**) and 3 biological replicates (**g**).

We have found that *M. loti* possesses a putative GA-synthetic operon. Our previous proteomic study showed the existence of an operon^15^, with symbiosis-specific expression (Fig. 1a). The operon exists on ‘symbiosis island’, which is horizontally-transferable chromosomal region existing in all rhizobia and contains many genes required for symbiosis such as nodulation (*nod*) genes and nitrogen fixation (*nif*) genes^16^,^17^,^18^. Real-time reverse transcription polymerase chain reaction (real-time RT-PCR) also validated the symbiosis-specific expression of the genes (Supplementary Fig. 1a). Thus, we hypothesized that these symbiosis-specific genes play some important roles in legume–rhizobia symbiosis. This operon was predicted to control GA synthesis because some rhizobia are reported to produce GA^19^,^20^, and four enzymes (encoded by mlr6368, mlr6369, mlr6370 and mlr6371) were confirmed to participate in the synthesis of *ent*-kaurene, a major GA intermediate, from dimethylallyl pyrophosphate and isopentenyl pyrophosphate (Supplementary Fig. 2)^21^.

Next, we found that GA produced by rhizobia negatively regulates the host nodule number. To investigate the effect of this putative rhizobial GA on the host plant, an *M. loti* signature-tagged mutagenesis (STM) mutant^22^ with a transposon insertion into mlr6368 (*gib*^−^ mutant) was inoculated into host *L. japonicus*, and the phenotypic changes were observed from 2 to 8 weeks post inoculation (WPI). In these plants, the number of days taken to flower, the length of the shoot and the number of rhizobial cells per plant were unchanged (Fig. 1b–d). However, from 4 WPI, nitrogen fixation activity per nodule and nodule weight significantly decreased, the nodule number significantly increased, and nodules formed at significantly lower position. (Fig. 1e–j). Host inoculated with STM mutants with a transposon insertion into other site of the operon also increased its nodule number (Supplementary Fig. 3). These results suggest that putative rhizobial GA is the symbiont-derived signal for regulation of nodule number to reject delayed infection and that the optimal nodule environment for nitrogen fixation is established by GA-associated regulation of the host nodule number.

### Identification of GA produced in nodule

**Figure 2:**
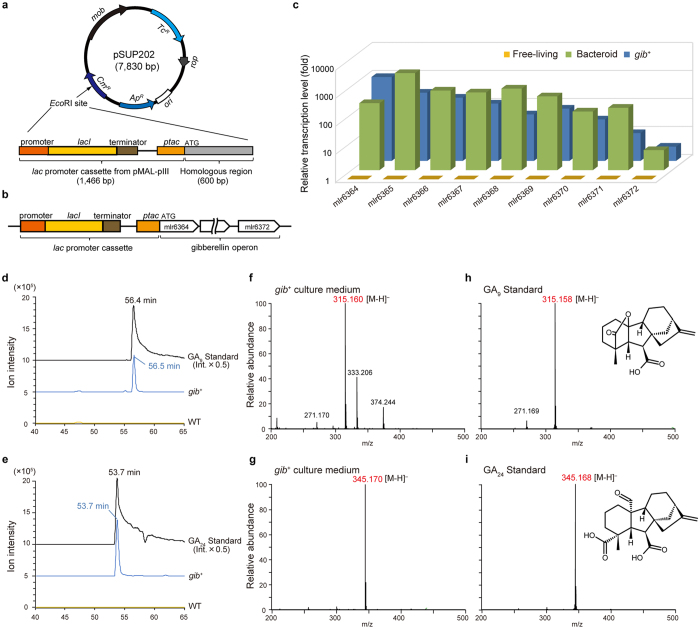
Construction of *gib*^*+*^ mutant and GA identification by LC–MS analysis. (**a**) Plasmid vector for homologous recombination of the *M. loti* genome. *Lac* promoter cassette in front of the homologous region (600 bp) is inserted into *Eco*RI restriction site. (**b**) *Lac* promoter insertion site. Note that *gib*^*+*^ mutant is a single-crossover mutant. (**c**) Relative expression levels of the operon genes. The fold-change values are relative to wild-type grown in liquid culture with 1 mM isopropyl β-D-1-thiogaractopyranoside (expression level = 1). Error bars indicate the standard error from 3 independent experiments. (**d,e**) NanoLC–MS chromatograms of culture medium of wild-type and *gib*^*+*^ mutant and authentic GAs. The chromatograms of mass range at m/z = 315.155–315.165 (**d**) and m/z = 345.165–345.175 (**e**) are extracted to focus on GA_9_ and GA_24_. (**f–i**) Mass spectra of *gib*^*+*^ culture medium at 56.5 min (**f**) and 53.7 min (**g**) and authentic GA_9_ (**h**) and GA_24_ (**i**). Insets show the structure of GA_9_ and GA_24_.

Candidate GAs produced by *M. loti* under symbiosis are numerous because >130 GAs have been found thus far^23^. To identify the rhizobial GA product, we constructed an *M. loti* mutant in which the operon was inductively expressed by the isopropyl β-D-1-thiogalactopyranoside (IPTG)-inducible promoter cassette in front of the gene operon (*gib*^+^ strain, Fig. 2a–c). After gene induction by 1 mM IPTG and cultivation for 24 h, the secreted products in liquid media were extracted with acidic ethyl acetate and analysed using liquid chromatography–mass spectrometry (LC–MS) equipped with a long monolithic column^15^. Spectral data revealed that the peaks of m/z = 315.16 [M-H]^−^ at 56.5 min and m/z = 345.17 [M-H]^−^ at 53.7 min were uniquely detected in the secreted product of the induced *gib*^+^ strain. These products were identified as GA_9_ and GA_24_ by comparison with authentic standards (Fig. 2d–i). GA_9_ and GA_24_ are both intermediates in GA synthesis in plants and fungi (Supplementary Fig. 4) and are produced by *M. loti* bearing a symbiosis-specific gene set.

**Figure 3:**
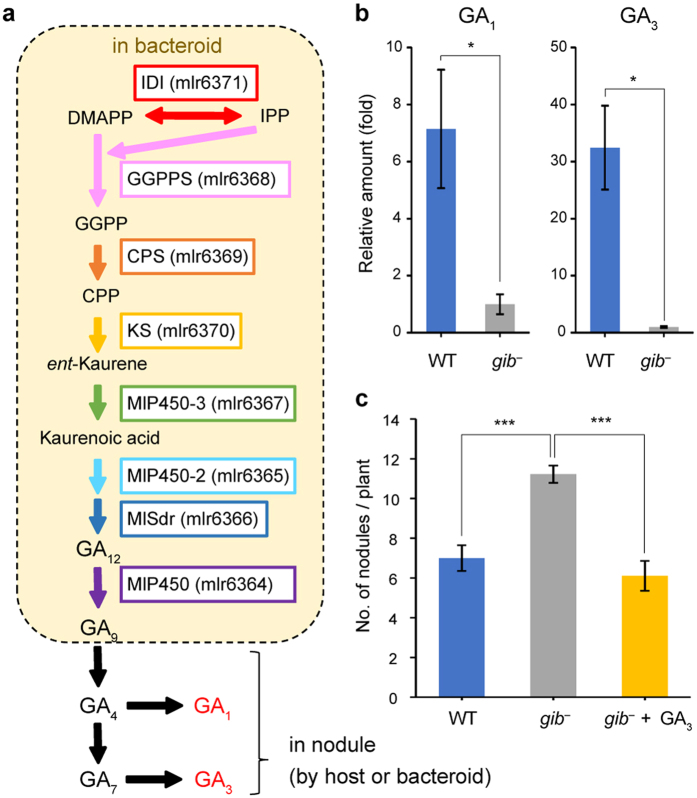
GA synthesis mechanism in nodules. (**a**) GA-synthetic pathway in nodules. Reactions mediated by enzymes encoded on the GA-synthetic operon are shown inside the broken-lined box. (**b**) The amount of GA_1_ and GA_3_ in plants inoculated with wild-type and *gib*^*−*^ mutant rhizobia were determined by LC–MS. GA_4_ and GA_7_ were below the detection limit under both conditions. Significant differences were determined using student *t*-test. *p < 0.05. Error bars indicate SEMs from 3 biological replicates. (**c**) Number of nodules. Water-dissolved GA_3_ was exogenously added at 10^−7^ mole per Incu Tissue at 2WPI. Multiple comparisons were corrected using the Holm-Bonferroni *t*-test. ***p < 0.001. WPI, weeks post inoculation. Error bars indicate SEMs from 9 plants.

Next, we sought to identify the GA-synthetic pathway in *M. loti*. Although plants and fungi synthesize various GAs using various pathways^24^, GA_9_ and GA_24_ are synthesized by almost similar pathway (Supplementary Fig. 4) and we predicted that the pathway used by *M. loti* would be similar. To identify the pathway, lysates from *Escherichia coli* expressing each of the functionally unknown 4 enzymes (encoded by mlr6364–mlr6367) were assayed *in vitro* with potential substrates. Three of the 4 enzymes were cytochrome P450s, and the other was a short-chain dehydrogenase reductase. For convenience, we named these enzymes MlP450-1 (mlr6364), MlP450-2 (mlr6365), MlSdr (mlr6366) and MlP450-3 (mlr6367). The products were analysed by gas chromatography–mass spectrometry (GC–MS) (Supplementary Fig. 5). MlP450-3 converted *ent*-kaurene into *ent*-kaurenoic acid (KA), indicating that it functions as an *ent*-kaurene oxidase. Assays of MlP450-2 and MlSdr together converted KA into GA_12_, whereas MlP450-2 or MlSdr alone did not. This result indicates that GA_12_ is synthesized from KA by the cooperative function of MlP450-2 and MlSdr, although KA oxidases from plants and fungi enzymatically convert KA into GA_12_. MlP450-1 converted GA_12_ into GA_9_ through GA_15_ and GA_24_, indicating that MlP450-1 functions as a GA20-oxidase. These results show that *M. loti* synthesizes GA_9_ via a pathway similar to that of higher plants and fungi with a slightly different enzymatic function (Fig. 3a, inside the broken-line box).

How do rhizobia regulate nodulation by GA_9_? Although GAs are major phytohormones that affect various plant development events, including nodulation, GA_9_ is one of the inactive GAs in higher plants. Only 3-hydroxylated GAs (GA_1_, GA_3_, GA_4_ and GA_7_) are known to be bioactive in higher plants, so we predicted that rhizobial GA_9_ was converted into bioactive GAs in nodules using the GA-synthetic pathway. *In vitro*, GA_9_ incubated with fractured nodule was converted into GA_1_ and GA_3_ via GA_4_ (Fig. 3a; Supplementary Fig. 6). GA_1_ and GA_3_ were elevated in plants inoculated with wild-type *M. loti* and not with the *gib*^*−*^ mutant (Fig. 3b). Therefore, we concluded that rhizobial GA_9_ functions in the host through its conversion into GA_1_ and GA_3_ mediated by the cooperative action of host and symbiont enzymes. Moreover, addition of GA_3_ to host with *gib*^*−*^ mutant decreased the nodule number (Fig. 3c).

### Distribution of GA synthetic genes

**Figure 4:**
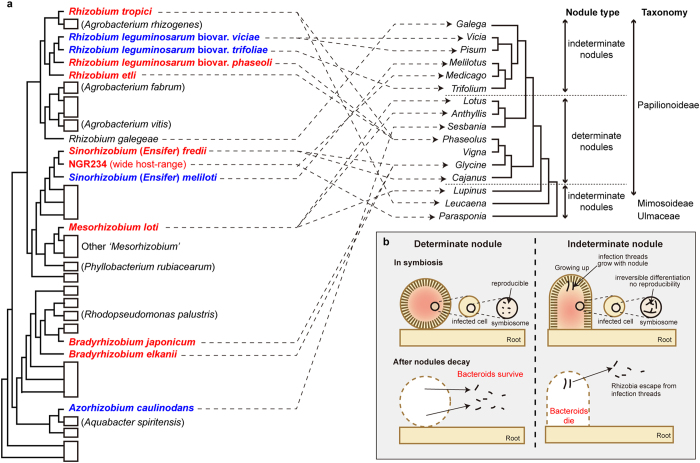
Phylogenies of legumes and bacteria, nodule types and distribution of GA synthetic genes. (**a**) Phylogenetic trees are modified from Doyle^43^. The bacterial phylogeny (left) is based on the 16S rRNA gene sequence. Representative symbiotic bacteria are shown; lineages of non-symbiotic bacteria are indicated by boxes. The bacteria possessing putative GA synthetic genes are shown in red bold characters and the bacteria without are shown in blue bold characters. Whether a bacterial species has GA-synthetic genes was determined by the presence of both *ent*-copalyl pyrophosphate synthase and *ent*-kaurene synthase by BLAST search. The phylogenetic relationships of legumes (and some non-legumes) based on *rbcL* sequence (right) are indicated for selected legume genera nodulated by bacteria that are shown on the bacterial tree. Nodule type and taxonomy are shown to the right of the tree. Arrows connect bacterial symbionts with their plant host. The wide host-range of NGR234 is shown by its ability to nodulate *Parasponia*. (**b**) Nodule structure of determinate and indeterminate nodules. Rhizobia lose the ability to reproduce after differentiation into bacteroids in indeterminate but not determinate nodules. Rhizobia typically possess the gibberellin operon only in hosts with determinate nodules.

To investigate the gene distribution among various rhizobial species, we performed BLAST (http://blast.ncbi.nlm.nih.gov/Blast.cgi) homology search against the genes in GA operon. We searched amino acid sequence similarities against two key genes for GA synthesis; *ent*-copalyl pyrophosphase synthase (mlr6369) and *ent*-kaurene oxidase (mlr6370). We chose these genes because *ent*-kaurene synthesis is powerful indicator for gibberellin synthesis and soil bacteria have some cytochrome P-450s irrelevant to GA syntehsis^25^. Interestingly, the genes for GA synthesis are found in species of rhizobia that inhabit the host in determinate nodules, including *M. loti*, *Bradyrhizobium japonicum*, *Sinorhizobium* (*Ensifer*) *fredii* and *Rhizobium etli.* Such genes are not found in rhizobial species, such as *S*. *meliloti* and *R*. *leguminosarum* biovar. *viciae*, which construct indeterminate nodules (Fig. 4, Supplementary Table 1). Among *R. leguminosarum*, GA synthetic genes were found only in *R. leguminosarum* biovar. *phaseoli*, but not in *R. leguminosarum* biovar. *viciae* and *R. leguminosarum* biovar. *trifoliae* although they belong to the same species. This result indicated that existence of GA-synthetic genes strongly correlated with the host nodule type.

### Competition assay of wild-type and *gib* ^−^ mutant

**Figure 5:**
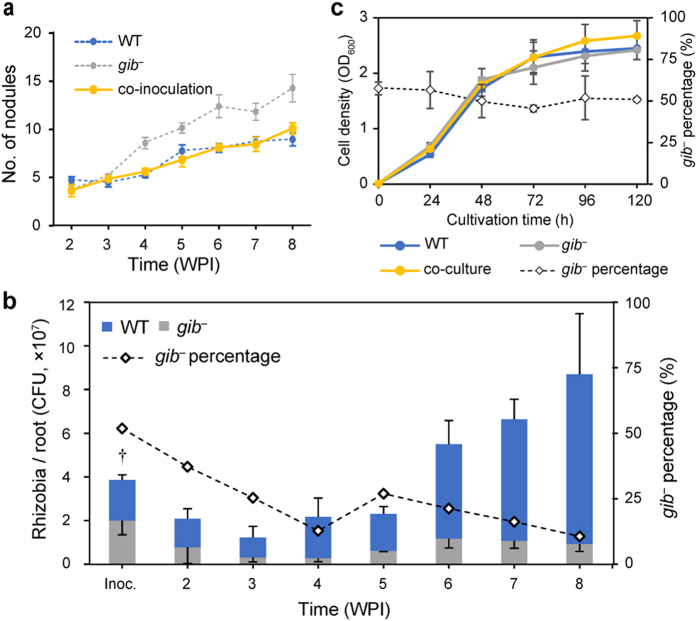
Competition between *M. loti* WT and *gib*^*−*^. (**a**) Number of nodules in co-inoculation assays. Nodule numbers for *gib*^*−*^ are shown in grey, taken from Fig. 1f. Error bars represent SEMs from > 8 plants. (**b**) The number and percentage of rhizobia in roots. The number above each bar indicates the percentage of *gib*^*−*^ colonies compared with the total colonies. Error bars indicate the SEMs from 3 plants. †: at this point, bacteria are isolated from soil (1 g) immediately after co-inoculation of WT and *gib*^*−*^. (**c**) Co-cultivation assay in liquid medium. Error bars indicate the SEMs from 3 independent experiments.

GA-synthetic genes are held by the rhizobia which construct determinate nodules (Fig. 4a). However, GA-synthetic genes are not necessary for symbiosis with host forming determinate nodules, because *gib*^*−*^ mutant which lacks the function of GA synthesis could infect into *L. japonicus* (Figs 1d–f and 3b,c). Therefore, we hypothesized that possession of GA-synthetic genes bring some advantages over non-possessor of the genes. To demonstrate the advantage of possessing GA-synthetic genes, wild-type and *gib*^*−*^ strains were used in competition assays. Co-inoculation of equal amount of wild-type and *gib*^*−*^ mutants demonstrated that the *gib*^*−*^ mutant gradually decreased in the population, without increasing nodule number (Fig. 5a,b), although such co-culture in liquid medium did not show this result (Fig. 5c) and individual inoculation assays of wild-type and *gib*^*−*^ mutants showed similar patterns of increasing numbers of rhizobial cells (Fig. 1d). These results show that possession of GA-synthetic genes is beneficial for growth in nodule.

## Discussions

In this study, we showed the phytohormonal regulation of nodule number from already-incorporated rhizobia in legume-rhizobia symbiosis. Nod factors (NFs) or exopolysaccharides (EPS) are symbiont-derived signals for nodulation^26^,^27^, however, rhizobial GA-dependent regulation of nodule number is different from the signals of NFs. Rhizobial GA regulates the nodule number under symbiotic condition, in contrast, NFs mainly initiate the nodulation process^28^. NFs are produced in response to flavonoid secreted from legume root hair, then induce the host nodule formation. There is a report that *nodC and nodD* genes were expressed during nodulation in *S. meliloti*^29^. It may play some roles in nodule development, however, the function of these bacterial genes inside the nodule are still functionally unknown, even if the NF receptors MtNFP and MtLYK3 were shown to be produced and required for bacterial release in the nodule, as Moling *et al*.^30^ mentioned. Okazaki *et al*.^31^ showed that *Bradyrhizobium elkanii* used type III secretion system to promote symbiosis by independent pathway of NF-dependent manner. This system also affects nodule number by activating host symbiosis signalling, however it is used when free-living rhizobia infect into host. Tian *et al*. found that already-incorporated *Sinorhizobium meliloti* inhibited the formation of infection thread by using cAMP signalling cascade^32^. This study showed that rhizobia played an active role in the control of infection, however, defective mutant of cAMP signalling did not show the significant difference in nodule number. Thus we conclude that regulation of nodule number by rhizobial GA is a new regulation mechanism which benefits the already-incorporated rhizobia.

Then what is the meaning of negative regulation of host nodule formation by rhizobia? Results of time-course nodulation assay (Fig. 1f) showed that rhizobial GA decreased the nodule number at 4 WPI when primary nodules developed into a mature state, and nodule formed at lower position in *gib*^*−*^ mutant (Fig. 1h). Lower nodule positions in *gib*^*−*^ indicate that nodules were formed at younger portion of root, i.e. delayed nodule formation. These results suggest that rhizobia in mature nodules prevented the delayed nodule formation. This hypothesis is supported by our previous quantitative proteomic data^33^, showing that the amount of protein produced through the operon gradually increased during nodule maturation (Supplementary Fig. 1b). Evolutionarily, the acquisition of nodule number control by rhizobia is presumed to be driven by the obstructive effects of delayed nodule formation by the following reasons: (i) too many nodules result in the decreased distribution of the carbon source per nodule from the host and (ii) rhizobia infecting the host after primary nodule maturation differ in genotype from those in primary nodules^34^. Thus, we considered that already-incorporated rhizobia are likely to reject delayed infection by other rhizobia, leading to the evolutionary acquisition of nodule number regulation.

How rhizobia regulate the host nodule number by synthesizing GA? Previous report showed that exogenously-added GA_3_ decreased the nodule number in *L. japonicus*^14^. The root hair curling triggered by rhizobia was inhibited by exogenous GA treatment. Exogenously added GA suppressed gene expression of nodule signalling pathway 2 (*NSP2*), which functions upstream of *NIN*, a gene required for the onset of nodule development^35^. In this study, the expression level of *NSP2* in hosts inoculated with *gib*^*−*^ *M. loti* was just slightly higher than that in hosts with wild-type *M. loti* at 5 WPI (Supplementary Fig. 7) like Maekawa *et al*.^14^. The result suggests that rhizobial GA inhibits infection of other rhizobia via similar pathway which exogenous GA does. On the other hand, the relationship between rhizobial GA-dependent regulation of nodule number and AON is still obscure. In *P. sativum,* regulation of nodulation by host GA is predicted to act independently of AON^12^. If it is applicable to *Lotus japonicus*, regulation by rhizobial GA also predicted to be independent from AON.

Then why are GA-synthetic genes distributed among rhizobia which inhabit determinate nodule? The answer to this question lies in the developmental process of determinate and indeterminate nodules. Within indeterminate nodules, bacteroids are deprived of duplication capability by nodule-specific, cysteine-rich peptides secreted by the host^18^,^19^. In contrast, bacteroids in determinate nodules still have the capacity for duplication, and they can escape from the nodule after the host plant dies (Fig. 4b)^36^. The characteristic of determinate nodule gives rhizobia advantage to reject delayed nodulation and to increase the population in nodule. Therefore, GA synthesis in rhizobia is presumably acquired in symbiont pairs involving a rhizobium and a host legume that does not kill its symbiont. This hypothesis of evolutionary acquisition of the genes is supported by the process of leguminous evolution. Now, the origin of nodulation is predicted to be approximately 60 million years ago, when all nodules were indeterminate nodules^37^. Determinate nodules arose from indeterminate-type ancestor in legume evolution, supporting that GA-synthetic genes were evolutionarily acquired after rhizobial symbiotic modules (symbiotic island or plasmid) were developed. Moreover, it was reported that the cytochrome P-450 cluster on NGR234 was 10% richer in G + C content than the rest of the symbiotic plasmid^17^, and had proteins with >80% similarity to those of other rhizobia (e.g. Supplementary Table 1), indicating that these genes have been selectively integrated into the symbiotic modules of rhizobia constructing determinate nodule. Furthermore, the micromolar concentrations of exogenously applied GA were reported to decrease the nodule number in *L. japonicus*, which forms determinate nodules^8^, but increase the nodule number in *P*. *sativum*, which forms indeterminate nodules^7^. These observations indicate that GA plays opposing roles in the formation of the two types of nodules. The negative effect of host with determinate nodule by GA allows rhizobia to decrease the nodule number.

Co-inoculation assay revealed that rhizobia which lacks GA synthesis were decreased gradually (Fig. 5b), although the growth rate in liquid medium was similar to wild-type (Fig. 5c). The mechanism of this phenomenon remains obscure, but we suggest that the rhizobial GA also affects rhizobial population in nodules. As many studies indicate plant-derived GA plays an important role in nodule formation reviewed in Hayashi *et al*.^9^ rhizobial GA might also affect proper nodule formation. Reduction of nitrogen fixing activity (Fig. 1e) and slightly smaller nodules in *gib*^*−*^ mutant (Fig. 1g) support this idea. In *gib*^*−*^ dominant nodule, nitrogen fixing activity maybe lower or nodule formation maybe slower than wild-type dominant nodule, then sanctioned by host plant when considering the sanction hypothesis^36^,^38^. Or rhizobial gibberellin may promote the bacterial release from the infection threads. Yet, this hypothesis have to be further experimentally proved.

**Figure 6:**
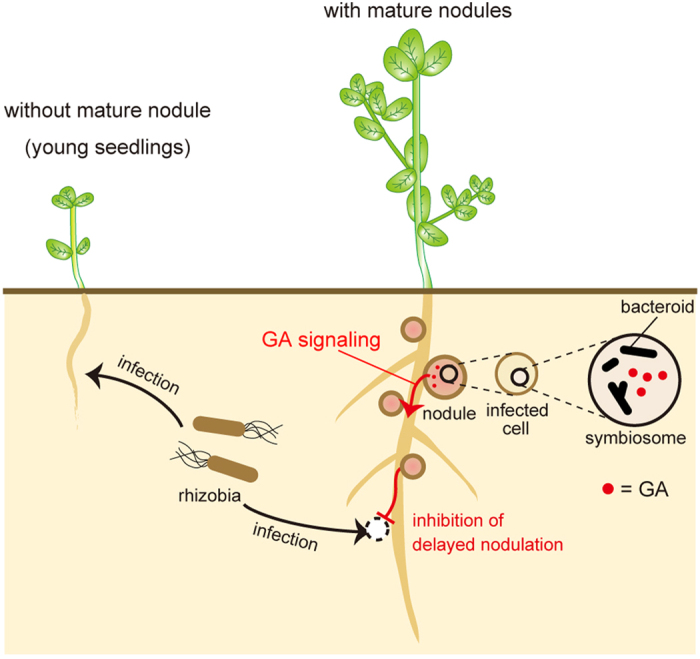
Model of nodule number regulation by rhizobial gibberellin. In the early developmental stage (no mature nodules), rhizobia inoculate into host plants only under host-derived control (e.g. auto-regulation of nodulation). After nodules have matured, the symbiont-derived regulation via rhizobial gibberellin also works to inhibit delayed infection by other rhizobia.

Here we show for the first time that *M. loti* synthesizes GA during symbiosis using enzymes unique to this condition and that synthesized GA maintains optimal nodule number and eventually optimizes the amount of nitrogen fixation per nodule. This finding is the first demonstration that a symbiont-derived hormonal signal regulates the host nodule number. These results enable us to propose a model of nodule number regulation by rhizobial GA (Fig. 6) and suggest that GA synthesis gives an evolutionary advantage for rhizobia in determinate nodules as it enables indigenous rhizobia to restrain delayed infection by other rhizobia. Our findings show the potential of symbionts to co-evolutionarily acquire the ability to regulate the host’s phenotype.

## Methods

### Strains, culture media and culture conditions

*M. loti* MAFF303099 and *L. japonicus* MG-20 Miyakojima^39^ were purchased from National Institute of Technology and Evaluation (Japan) and National BioResource Project (Japan), respectively. STM mutant of mlr6364 (Clone ID: 36T01f03), mlr6365 (Clone ID: 10T05d07), mlr6367 (Clone ID: 05T04e11), mlr6368 (Clone ID: 10T01h01 as *gib*^*−*^ mutant), mlr6370 (Clone ID: 01T03h03) and mlr6371 (Clone ID: 14T06b04) were purchased from National BioResource Project (Japan). Bacterial cells were grown in Trypton–Yeast extract (TY) medium at 28 °C. Phosphomycin (100 μg/mL), tetracyclin (5 μg/mL), spectinomycin (100 μg/mL), streptomycin (100 μg/mL) and isopropyl β-D-1-thiogaractopyranoside (IPTG; 1 mM) were added if needed. For nodule collection to perform fractured nodule assay, *L. japonicus* MG-20 Miyakojima seeds were sterilized, germinated and inoculated with *M. loti* and grown in MM1^40^ medium at 25 °C with a 16-h light/8-h dark cycle.

### Plant assays

*M. loti* Wild-type and STM mutants were cultured in TY medium with suitable antibiotics until the OD_600_ reached approximately 1.0. Cells were washed and resuspended in B & D nitrogen-free medium to an OD_600_ of 1.0, then 20 mL of bacterial suspension was inoculated at 3 week after *L. japonicus* seedlings were planted on vermiculite in Incu tissue (SPL Life Sciences, Korea). To measure the CFU (Fig. 1d), roots from each plant were homogenized in PBS and plated onto TY agar plates containing phosphomycin. Colonies were counted on the plates after 7 days of incubation at 28 °C. Nodule number was visually confirmed. For addition of GA_3_ (Fig. 3c), water-dissolved GA_3_ was exogenously added at 10^−7^ mole per Incu Tissue at 2WPI. Significant differences between two groups were determined by student *t*-test (Fig. 1b,c,g). Multiple comparisons were corrected using the Holm–Bonferroni *t*-test (Figs 1d–f and 3c). Nodule positions were measured using over 200 nodules from 20 (*gib*^*−*^) and 30 (WT) plants. Significant difference was determined by Mann-Whitney *U*-test.

### Genetic recombination of *M. loti*. 

Insertion of the *lac* promoter cassette in front of the gibberellin operon was performed using the suicide vector pSUP202 and primers (Supplementary Table 2). Six hundred base pair regions just upstream of the translation-initiating sequence of the target operon were amplified from *M. loti* genomic DNA using primers gibpromoter-f/r, and 1466 base *lac* promoter cassette were cloned from pMAL-pIII vector (New England Biolabs, MA, USA) with the primers lac-f/r. PCR products were then cloned into the pSUP202 EcoRI site using In-Fusion HD Cloning Kit (Takara Bio, Japan) and confirmed by sequencing. pSUP202 constructs were transferred from *Escherichia coli* strain S17-1 into *M. loti* by conjugation, and transconjugants were selected on TY+ phosphomycin (100 μg/mL) + tetracyclin (10 μg/mL) agar plate 3 times before confirmation by sequencing of genomic DNA.

### Isolation of RNA and preparation of cDNA

*M. loti* bacteroids were prepared using the method of Uchiumi *et al*.^16^. Total RNAs were extracted from bacterial cells at log phase (OD_600_ = 1.0), and 50 mg of plant roots using the RNeasy Mini Kit (QIAGEN, Hilden, Germany) or RNeasy Plant Mini Kit (QIAGEN), respectively, according to the manufacturer’s protocol. cDNA synthesis was performed using a High Capacity DNA Reverse Transcription kit (Applied Biosystems by Life Technologies, Carlsbad, CA, USA) with 2 μg of the total RNAs as a template. The reaction was performed according to the manufacturer’s protocol.

### Quantitative real-time RT-PCR analysis

For the quantitative PCR, *sigA* (mll2466) or *LjATPS* (ATP synthase) was used as an endogenous control for bacteria or plants to normalize the expression data for each gene, respectively. The primers (Supplementary Table 2) were designed using Primer Express software (Applied Biosystems, Foster City, CA). Amplification was performed using Power SYBR Green PCR Master Mix (Applied Biosystems) in the 7500 Real-Time PCR System (Applied Biosystems). The reporter signals were analysed using the 7500 Real-Time PCR System.

### Identification and quantification of GAs by LC-MS

From the 10 mL of *M. loti* (WT and *gib*^+^) culture supernatant, organic compounds were extracted using equal volumes of ethyl acetate (EtOAc) thrice after acidification to pH 3 by HCl. The organic phase was collected, dried by evaporation and dissolved in 100 μL of water containing 1% acetic acid. Approximately 50 mg of nodules was ground in 3 mL of 80% (v/v) acetone containing 1% (v/v) acetic acid, and incubated for 24 h at 4 °C and then centrifuged at 3000 × *g* for 20 min at 4 °C. The supernatant was concentrated to dryness, dissolved in 0.5 mL of water containing 20% methanol and 1% acetic acid and analysed by LC–MS. Exact mass analysis was performed using a nano-LC (Ultimate 3000, DIONEX)–MS system (LTQ Orbitrap Velos, Thermo Fisher Scientific). Extracts (5 μL) were injected and separated by reversed-phase chromatography using a monolithic column^41^ (2000 mm, 0.1 mm I.D., Kyoto Monotech Co., Ltd., Kyoto) at a flow rate of 1.0 mL/min. The gradient was 10% (0–10 min), 10–99% (10–40 min) and 99–100% (40–45 min) 80% acetonitrile/water containing 0.1% formic acid in water containing 0.1% formic acid. The MS was operated with an ESI voltage of 2.3 kV and a transfer tube temperature of 280 °C. MS data acquisition was set from 20 to 65 min. The chromatograms of mass range at m/z = 315.155–315.165 (Fig. 2d) and m/z = 345.165–345.175 (Fig. 2e) are extracted to focus on GA_9_ and GA_24_.

### Plasmid construction and protein production in *E. coli*

The full-length coding sequences of 8 genes were amplified using each primer pair (Supplementary Table 2). The PCR products were cloned into the pET-21a expression vector (Novagen) using the In-Fusion HD Cloning Kit (Takara Bio) to generate fusions with 6-His-tags at the C termini. The vectors were then introduced into *E. coli* cells strain BL21 Star (DE3) (Stratagene). For protein production, the culture (5 mL) was incubated at 37 °C to OD 0.5 at 600 nm. The temperature was then decreased to 16 °C, and IPTG (0.1 mM) was added. The culture was incubated for 24 h and then centrifuged at 4 °C. The pellet was washed using phosphate buffer (100 mM, pH 7.0) and then resuspended in lysis buffer (50 mM HEPES pH 7.4, 10% (w/v) glycerol, 5 mM MgCl_2_). The samples were sonicated and the supernatants were used for the reactions. Protein concentration was measured by Protein Assay Bicinchoninate Kit (Nacalai Tesque, Kyoto, Japan)

### Reaction conditions of enzyme assays

For functional validation of mlr6uchi mlr6369, mlr6370 and mlr6371, *in vitro* enzyme assays were performed in the following buffer: 50 mM HEPES (pH7.4), 10% (w/v) glycerol and 5 mM MgCl_2_. For each assay, 100 μL of *E. coli* lysate (containing 10 mg/mL protein) was incubated in 0.5 mL buffer with 20 μM of each substrate [isopropyl pyrophosphate, dimethylallyl pyrophosphate, geranyl pyrophosphate (GPP), farnesyl pyrophosphate, and geranyl GPP; Sigma-Aldrich] and incubated at 37 °C for 6 h. The reaction products were dephosphorylated by adding 20 units of alkaline phosphatase (intestinal calf; New England Biolabs) for 1 h at 37 °C. Terpene alcohols produced by dephosphorylation were then extracted with an equal volume of hexane. The organic phase was then dried, resuspended in hexane and analysed using a GC–MS-QP2010 Ultra (Shimadzu, Japan) in electron ionization (70 eV) mode, equipped with a 30 m × 0.25-mm diameter with 0.25 μm film of HP-1 MS column (Agilent Technologies, Santa Clara, CA). Samples (1 μL) were injected in splitless mode at 50 °C. After holding for 3 min at 50 °C, the oven temperature was raised at a rate of 14 °C/min to 300 °C, where it was held for an additional 3 min. MS data were collected by selected-ion monitoring mode focused on m/z corresponding to each product. Geranylgeraniol (GGOH) and *ent*-kaurene were identified by comparison of their mass spectra retention times with authentic standards (GGOH, Sigma Aldrich; *ent*-kaurene, Olchemim Ltd., Czech Republic). Selected ion monitoring (SIM) mode were used to draw the chromatogram of GGOH and *ent*-kaurene. SIM parameters were set at m/z 290 for GGOH, and 272 for *ent*-kaurene.

For the functional validation of MlP450-1,2,3 and MlSdr, *in vitro* enzyme assays were performed using the following procedure. *E. coli* lysates (100 μL) were mixed with 3.5 μM spinach ferredoxin, 0.1 U spinach ferredoxin reductase, 2 mM NADPH and 2 mM NADP^+^ (Simga-Aldrich) in a final volume of 1 mL of 100 mM Tris-HCl, pH 7.5. Substrates were added at a final concentration of 100 μM, and the reaction was incubated overnight at 30 °C. Control incubations were performed with the addition of *E. coli* lysates bearing the empty pET21a vector. The reactions were stopped by adding 50 μL HCl and 1 mL EtOAc and centrifuged at 3000 rpm for 10 min. Kaurenoic acid and gibberellic acids were methylated using trimethylsilyldiazomethane (TMS, Nacalai Tesque) in toluene containing 20% methanol. Samples were then trimethylsilylated using N-methyl-N-(trimethylsilyl)trifluoroacetamide for addition to hydroxyl groups. GC–MS analysis was performed as described in bacteroid assays using authentic standards (GA_3_, Sigma-Aldrich; Kaurenoic acid, GA_1_, GA_4_, GA_7_, GA_9_, GA_12_, GA_15_ and GA_24_; Olchemim Ltd.).

### Reactions using fractured nodules

Isolated nodules (1 g) were homogenized in a mortar with extraction buffer (10 mL, 50 mM KHPO4 pH 7.0, 200 mM sodium ascorbate and 1.2 g polyvinyl pirrolidone). The homogenate was centrifuged at 8500 × g for 20 min, washed and resuspended in reaction buffer (50 mM TES pH 7.5, 2.5 mM MgCl_2_ and 1 mM KHPO_4_). Substrates (100 μM) were then added to the 1-mL suspensions and incubated for 48 h at 28 °C with shaking at 200 rpm. For product extraction, suspensions were extracted with an equal volume of EtOAc after acidification to pH 3.0. The extracts were methylated using TMS-diazomethane and measured by GC–MS as described above. SIM parameters were set at m/z 418, 506, 416 and 504 to detect GA_4_, GA_1_, GA_7_ and GA_3_, respectively.

### Acetylene reduction assay

The whole-plant nitrogenase activity was determined by acetylene reduction assay. An individual plant was packed into a 5-mL headspace vial (GL Science, Tokyo, Japan) in which 20% of the air was replaced by acetylene gas generated by CaC_2_ (Sigma-Aldrich). After a 3-h reaction at 25 °C, the atmosphere (1 mL) was injected into a GC–MS (30 m × 0.32-mm diameter HP-PLOT Q column with 0.20 μm film) (Agilent Technologies) at 40 °C, followed by a gradient from 40 °C to 80 °C at 5 °C/min and then from 80 °C to 180 °C at 50 °C/min.

### Co-inoculation assays

Wild-type and *gib*^*−*^ were cultured in TY + phosphomycin medium until the OD_600_ reached approximately 1.0. Cells were then washed and resuspended in B & D nitrogen-free medium^42^ to an OD_600_ of 1.0. Both wild-type and *gib*^*−*^ suspensions were mixed, and 20 mL of bacterial mixture was inoculated into the culture. To measure the CFU, roots from each plant were homogenized in PBS and plated onto TY agar plates containing phosphomycin with or without streptomycin and spectinomycin. Colonies were counted on the plates after 7 days of incubation at 28 °C. The percentage of *gib*^*−*^ in the population was calculated as (output streptomycin and spectinomycin-resistant colony count)/(output of all colony count).

### Phylogenetic trees

The phylogenetic trees were referred from Doyle^43^. The bacterial phylogeny which was based on the 16S rRNA gene sequence (Supplementary Table 3) was constructed by neighbour-joining method using Clustal X (http://www.clustal.org/). The parameters were set to 111 for ‘random number generator seed’ and 1000 for ‘number of bootstrap trials’. Whether a bacterial species has GA-synthetic genes was determined by the presence of both *ent*-copalyl pyrophosphate synthase (mlr6369) and *ent*-kaurene synthase (mlr6370) by BLAST search (Supplementary Table 1). On the other hand, phylogenetic relationships of legumes (and some non-legumes) which are based on *rbcL* sequence (Supplementary Table 4) were also constructed using Clustal X.

## Additional Information

**How to cite this article**: Tatsukami, Y. and Ueda, M. Rhizobial gibberellin negatively regulates host nodule number. *Sci. Rep.* **6**, 27998; doi: 10.1038/srep27998 (2016).

## Supplementary Material

Supplementary Information
