# Supplementary material for: Rhizobial gibberellin negatively regulates host nodule number
Source: Sci Rep. 2016 Jun 16;6:27998. doi: 10.1038/srep27998 (PMC4910070; doi:10.1038/srep27998)
Supplement: Supplementary Information [file srep27998-s1.pdf]

Supplementary Information

Rhizobial gibberellin negatively regulates host nodule number

Yohei Tatsukami[1,2], Mitsuyoshi Ueda[1]

[1]Division of Applied Life Sciences, Graduate School of Agriculture, Kyoto University, Sakyo-ku, Kyoto 606-8502, Japan

[2]Japan Society for the Promotion of Science, Sakyo-ku, Kyoto, Japan

Corresponding author: Mitsuyoshi Ueda

(Email: miueda@kais.kyoto-u.ac.jp, TEL/FAX: +81-75-753-6112)

Supplementary Figure 1

a

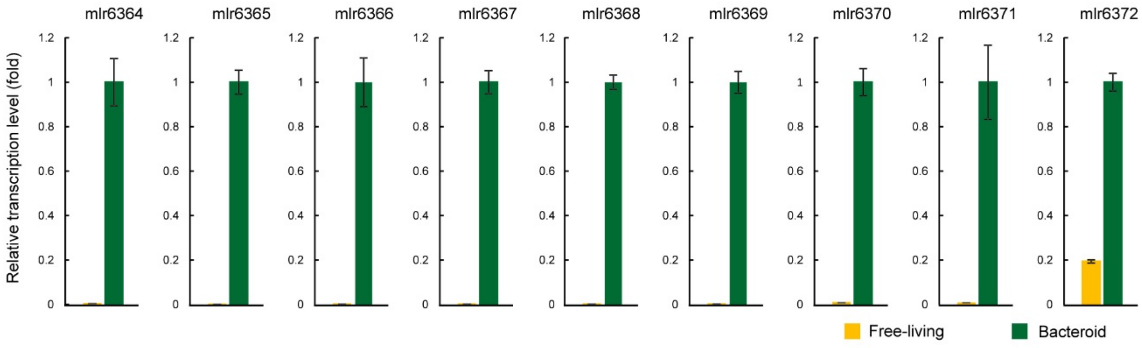

b

Accession number	2WPI	3WPI	4WPI
mlr6364	1 ± 0.36	1.85 ± 0.20	2.43 ± 0.27
mlr6365	1 ± 0.07	2.06 ± 0.36	2.38 ± 0.16
mlr6366	1 ± 0.13	1.59 ± 0.49	1.49 ± 0.26
mlr6369	1 ± 0.20	1.68 ± 0.09	3.09 ± 0.27
mlr6371	1 ± 0.03	2.19 ± 0.26	3.92 ± 0.39

Supplementary Figure 1. Symbiosis-specific function of the GA-synthetic operon.
(a) Relative expression levels of the operon genes. The fold-change values are relative to *M. loti* bacteroids at 4 weeks post-inoculation (WPI) (expression level = 1). Error bars indicate the SEMs from 3 independent experiments. (b) Protein expression levels from previous quantitative proteomic studies.[16] Expression levels are relative to *M. loti* at 2 WPI (expression level = 1). Values show mean ± SEM.

Supplementary Figure 2

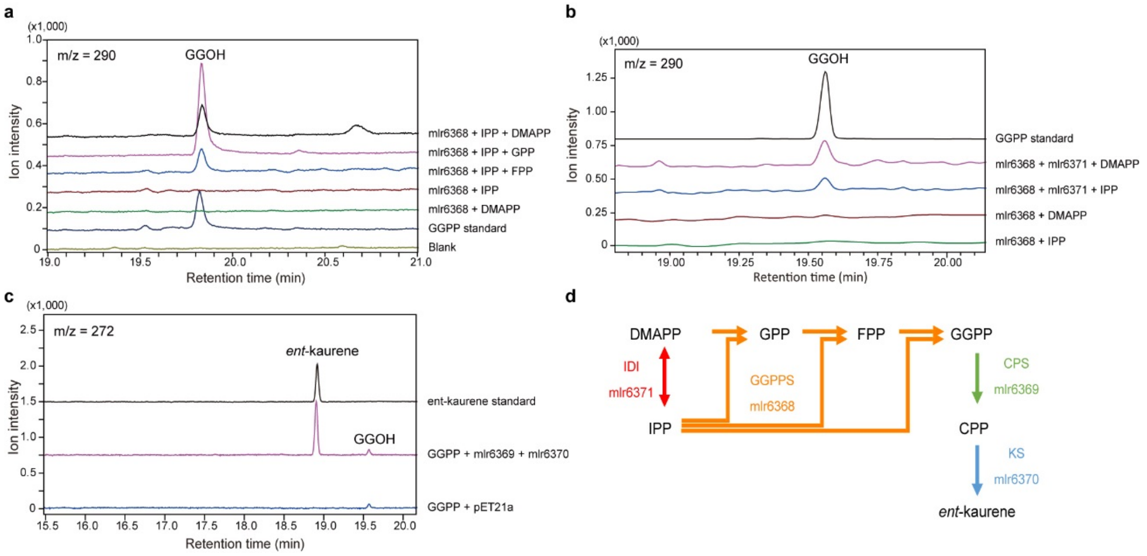

Supplementary Figure 2. *In vitro* confirmation of the enzymes encoded by mlr6368, mlr6369, mlr6370 and mlr6371. (a, b) GC–MS analysis (SIM; m/z = 290) of dephosphorylated products resulting from *in vitro* assays using prenyl alcohols as a substrate and lysates isolated from mlr6368-expressing *E. coli* (a) and mlr6371-expressing *E. coli* (b). (c) GC–MS analysis (SIM; m/z = 272) of *ent*-kaurene rom *in vitro* assays using GGPP as a substrate and lysates isolated from mlr6369- and mlr6370-expressing *E. coli*. (d) The confirmed *ent*-kaurene synthetic pathway reported by Hershey *et al*., 2014 (mlr6369 and mlr6370) and predicted by genetic information (mlr6368 and mlr6371). Note that GGPP was dephosphated to GGOH by alkaline phosphatase to detect by GC-MS, because GGPP is hard to be detected by GC-MS. GGOH, geranylgeraniol; DMAPP, dimethylallyl pyrophosphate; IPP, isopentenyl pyrophosphate; GPP, geranyl pyrophosphate; FPP, farnesyl pyrophosphate; GGPP, geranylgeranyl pyrophosphate; CPP, *ent*-copalyl pyrophosphate; IDI, isopentenyl-diphosphate isomerase; GGPPS, geranylgeranyl pyrophosphate synthase; CPS, *ent*-copalyl pyrophosphate synthase; KS, *ent*-kaurene synthase.

Supplementary Figure 3

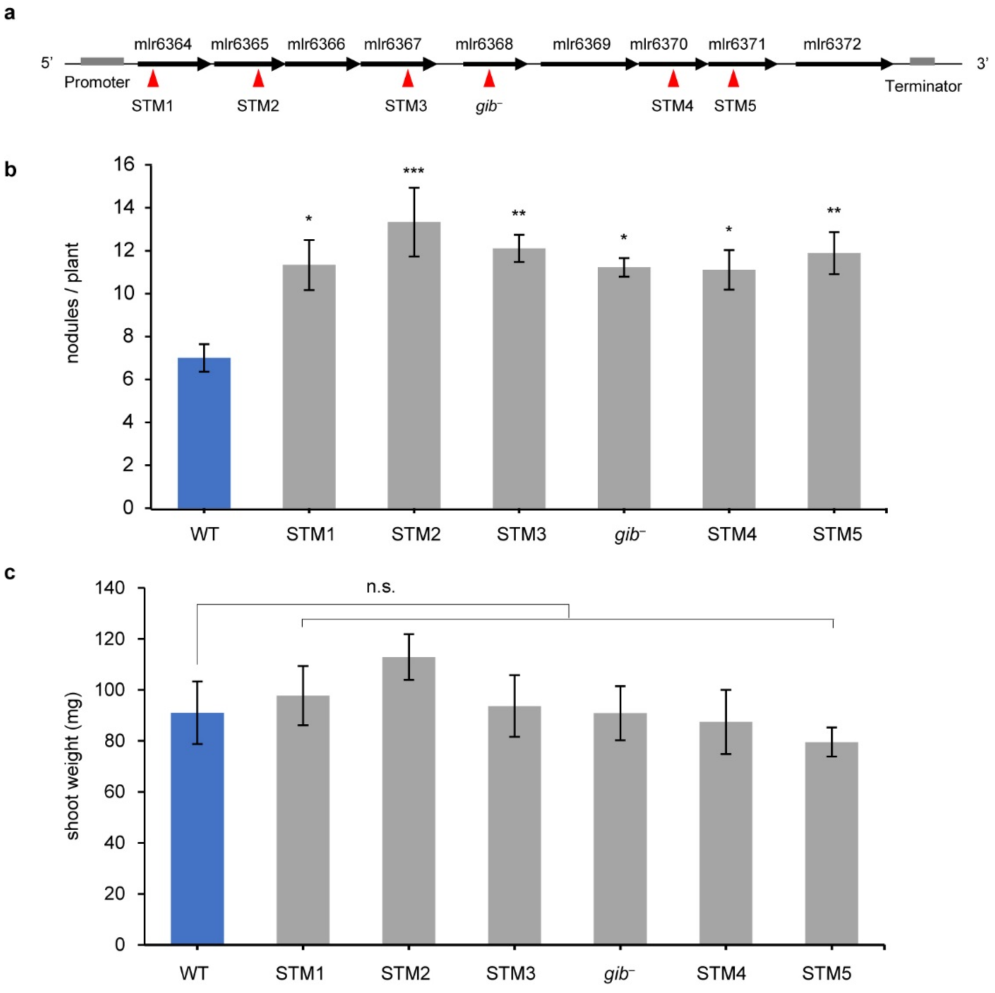

Supplementary Figure 3. Inoculation assay with STM mutants. (a) The operon consists of 9 genes with accession numbers mlr6364– mlr6372. The arrowheads indicate the transposon insertion site of each STM mutant. (b) Number of nodules and (c) Shoot weight of the host plant were measured at 5WPI. Multiple comparisons were corrected using the Dunnett's test. *p < 0.05, **p < 0.01, ***p < 0.001. WPI, weeks post inoculation. Error bars indicate SEMs from 9 plants.

Supplementary Figure 4

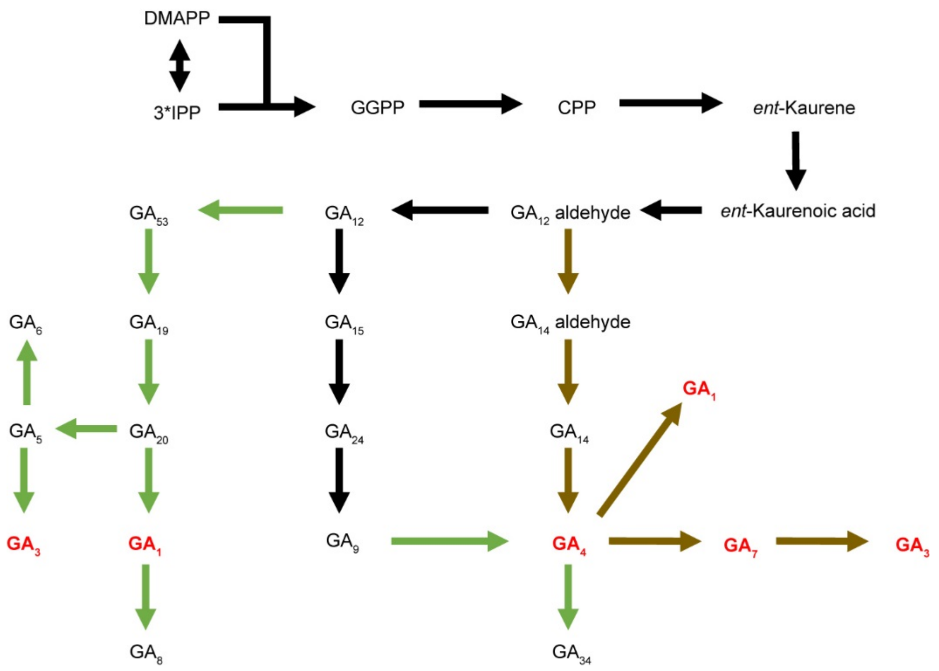

Supplementary Figure 4. Gibberellin biosynthetic pathway in higher plants and the fungus *Gibberella fujikuroi*. Bioactive GAs appear in red. The pathways existing commonly, only in the fungus, and only in higher plants are shown in black, brown, and green arrows, respectively. DMAPP, dimethylallyl pyrophosphate; IPP, isopentenyl pyrophosphate; GGPP, geranylgeranyl pyrophosphate; CPP, *ent*-copalyl diphosphate.

Supplementary Figure 5

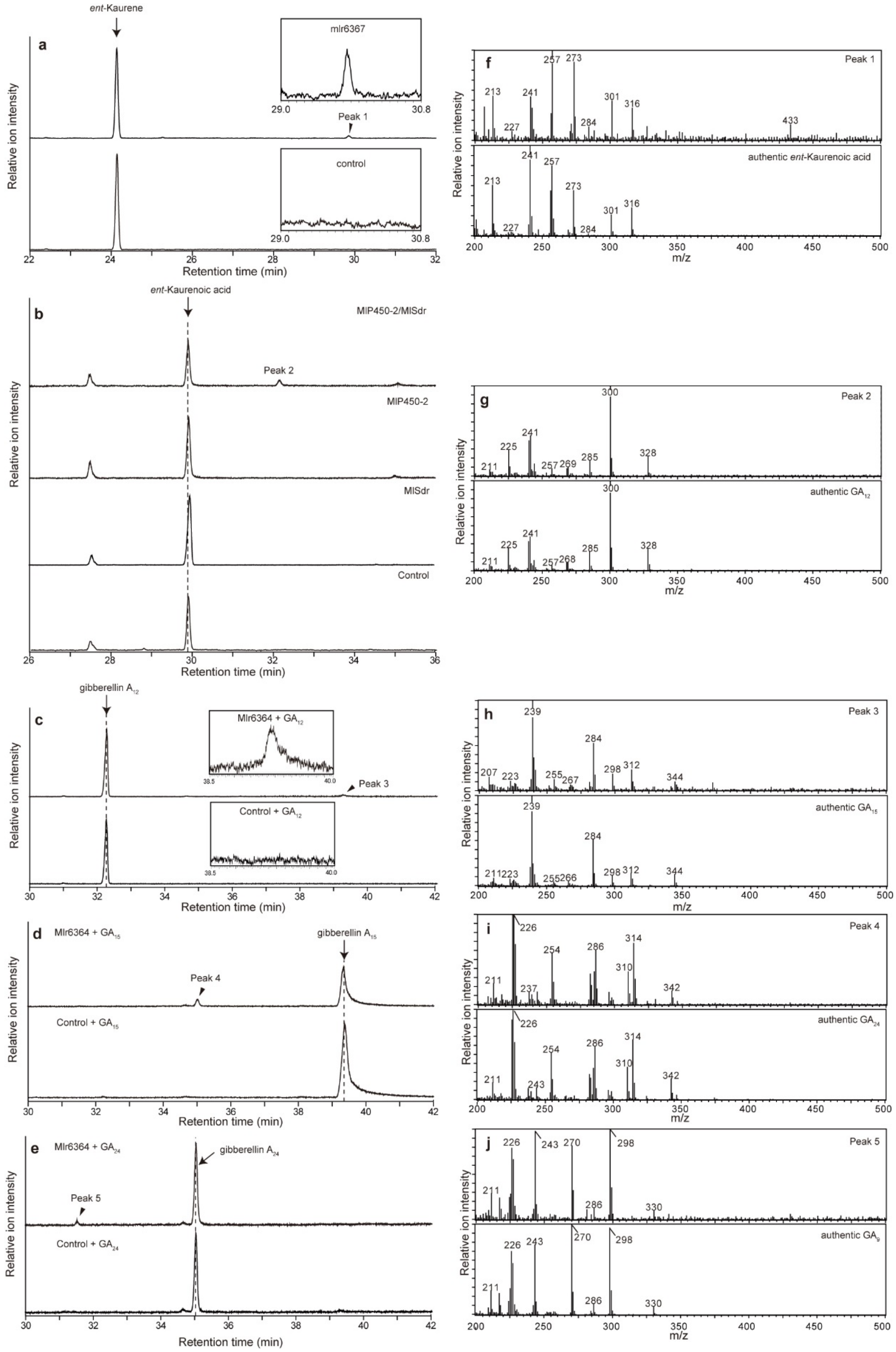

Supplementary Figure 5. *In vitro* enzymatic assays of MlP450s and MlSdr. (a–e) Total ion chromatograms of reaction products from *in vitro* assays. (a) Reaction of *ent*-kaurene with lysates isolated from MlP450-3-expressing *E. coli* and empty-vector control *E. coli*. (b) Reaction of *ent*-kaurenoic acid with lysates isolated from MlP450-2 and/or MlSdr-expressing *E. coli* and empty-vector control *E. coli*. (c–e) Reaction of GA_{12} (c), GA_{15} (d) and GA_{24} (e) with lysates isolated from MlP450-1-expressing *E. coli* and empty-vector control *E. coli*. (f–j) Mass spectra of peaks 1–5 compared with authentic gibberellin or *ent*-kaurenoic acid.

Supplementary Figure 6

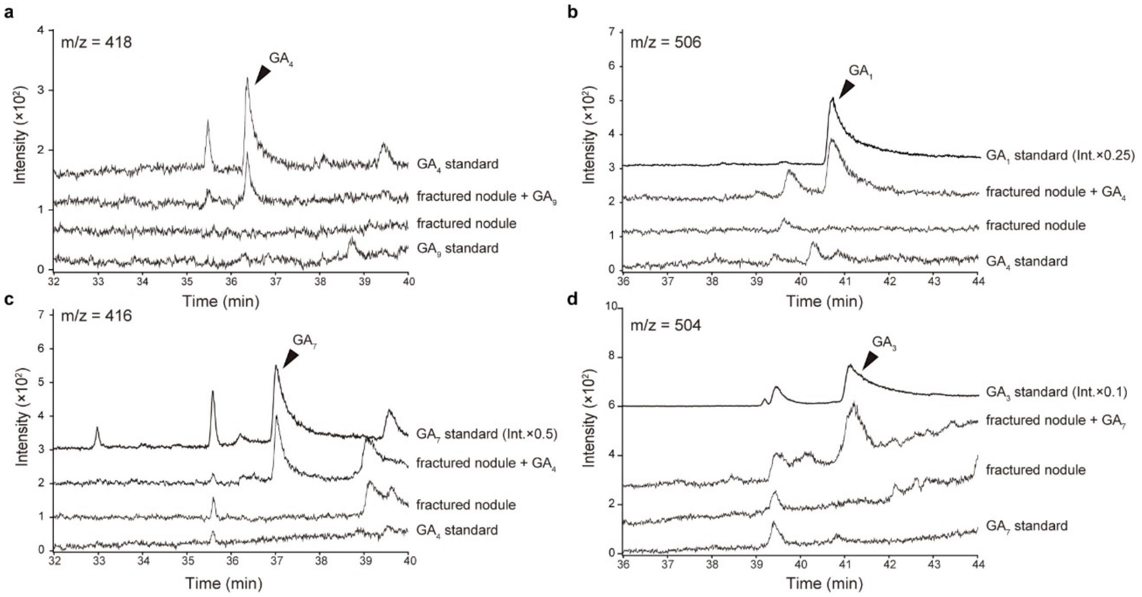

Supplementary Figure 6. Reactions using fractured nodules. Fractured nodules were incubated with substrate (GA_9, GA_4 or GA_7). GAs were extracted with acidic ethylacetate. Extracts were methylated and trimethylsilylated and measured by GC–MS. Selected ion monitoring modes of m/z = 418 (a), 506 (b), 416 (c) and 504 (d) were used to detect GA_4, GA_1, GA_7 and GA_3, respectively.

Supplementary Figure 7

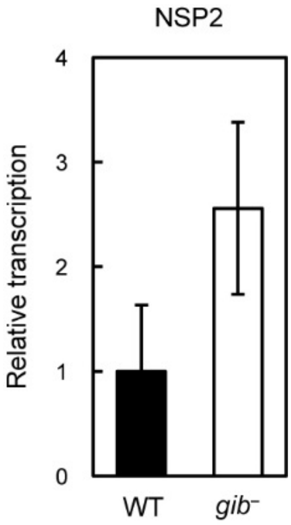

Supplementary Figure 7. Relative expression levels of NSP2. Fold-change values are relative to host inoculated with *M. loti* wild-type (expression level = 1) at 5 WPI. Error bars indicate the standard error from 3 plants.

Supplementary Table 1. Distribution of GA synthetic genes among rhizobia

species		ent-copalyl pyrohposphase synthase		ent-kaurene oxidase	
name	taxid	NCBI Reference Sequence ID	Homology (%)	NCBI Reference Sequence ID	Homology (%)
Mesorhizobium loti (reference)	381	WP_010913996.1	100	WP_010913997.1	100
Rhizobium tropici	398	WP_004120019.1	90	WP_004120017.1	88
Rhizobium leguminosalum bv. *viciae*	387	–	–	–	–
Rhizobium leguminosalum bv. *trifoliae*	386	–	–	–	–
Rhizobium leguminosalum bv. *phaseoli*	385	WP_037145548.1	86	WP_037145551.1	88
Rhizobium etli	29449	WP_040140636.1	88	WP_020923331.1	91
Sinorhizobium fredii	380	WP_014857759.1	95	WP_014857758.1	94
NGR234 (wide host-range)	394	WP_010875301.1	94	WP_010875302.1	94
Sinorhizobium meliloti	382	–	–	–	–
Bradyrhizobium japonicum	375	WP_026312707.1	93	WP_028153675.1	93
Bradyrhizobium elkanii	29448	WP_028350654.1	93	WP_028350655.1	94
Azorhizobium caulinodans	7	–	–	–	–

Supplementary Table 2. Primers used in this study

Primer	Sequence (from 5’ to 3’)
**plasmid construction**	
gibpromoter-f	CAGGAAACAGCCAGTATGTCCGAACAACCCTTGCCGAC
gibpromoter-r	TTTCATTGCCATACGCTTGTCCCACATCTTGCCGCG
lac-f	ATGAATGCTCATCCGTGGTGCAAAACCTTTCGCGGTATGG
lac-r	ACTGGCTGTTTCCTGTGTGAAATTGTTATCC
pET21a-mlr6364-f	AGAAGGAGATATACATATGTCCGAACAACCCTTGCCGAC
pET21a-mlr6364-r	GTGGTGGTGGTGGTGCCAGAGCACCGGGAACTCCTC
pET21a-mlr6365-f	AGAAGGAGATATACATATGGACGTGCAAGAAACCACGGC
pET21a-mlr6365-r	GTGGTGGTGGTGGTGGCCCCCCTGAGCATGCAGTC
pET21a-mlr6366-f	AGAAGGAGATATACATATGCGCGTCGTGATCGATCAGG
pET21a-mlr6366-r	GTGGTGGTGGTGGTGGCAGCGCGGCCCCGC
pET21a-mlr6367-f	AGAAGGAGATATACATATGGACATGCTGCTCAACCCGC
pET21a-mlr6367-r	GTGGTGGTGGTGGTGTGAGAATCCGATGCGGATTTTCATGGAC
pET21a-mlr6368-f	AGAAGGAGATATACATGTGAACGCGCTGTCCGAACAG
pET21a-mlr6368-r	GTGGTGGTGGTGGTGTGGCGCCGCTCCTGCTCC
pET21a-mlr6369-f	AGAAGGAGATATACATATGATCCAGACCGAACGCGCGC
pET21a-mlr6369-r	GTGGTGGTGGTGGTGGGCGGGCGCACGCTGG
pET21a-mlr6370-f	AGAAGGAGATATACATGTGGGCGGGGAGAAATCTCATAC
pET21a-mlr6370-r	GTGGTGGTGGTGGTGACGCCGGAAAAAGAACCGCATCG
pET21a-mlr6371-f	AGAAGGAGATATACATATGTCCGAACAACCCTTGCCGAC
pET21a-mlr6371-r	GTGGTGGTGGTGGTGCCAGAGCACCGGGAACTCCTC
**RT-qPCR**	
mlr2466-f	GCCAGATGCTGCACGAGAT
mlr2466-r	TTCAAGACCTTGCGCACTTTT
mlr6364-f	GAAAATCCGCACCTGACGTT
mlr6364-r	CGCCATGCCGATGCA
mlr6365-f	TGGACCTGCATTGGGAATTC
mlr6365-r	GAGCCCGAACATGTCATCCT
mlr6366-f	CATGCCGCGAAAGACCAA
mlr6366-r	GGTCACCACGGCCACCTT
mlr6367-f	CCTGGTATGGCTGGAAATGG
mlr6367-r	CCCCGGCCTTGTGCAT
mlr6368-f	GGCGCGTGCTTGAACAG

Primer	Sequence (from 5’ to 3’)
mlr6368-r	GGAACGGTTGGCGCAGAT
mlr6369-f	TCTGGCCGATCAATGTGTTC
mlr6369-r	GCCCGGCCAGATGCA
mlr6370-f	TGCACAACGGCATCGATT
mlr6370-r	AGCAACGACAGGCAACAGAA
mlr6371-f	TGGAGGCCGATGGACTCA
mlr6371-r	CCGGCTGTACCGCTTCCT
mlr6372-f	CGGAGCGCGCTTCACT
mlr6372-r	ACCGCGGGCAATTGCT
LjATPS-f	ACATGCTTGCACCATACCAA
LjATPS-r	TCCCCAACTCCAGCAAATAC
NSP2-f	CATCGACTCCATGATTGACG
NSP2-r	GGTTGTTGTTGTCGTGGTTG

Supplementary Table 3. Bacterial strains and sequences used for phylogeny

Name	Strain	Type strain (T)	Source of 16S rRNA sequence: NCBI RefSeq	Genbank
**Strains described in Figure 4**				
Rhizobium tropici	CIAT 889	T	R_102511.1	
Agrobacterium rhizogenes	IFO 13257	T	NR_043398.1	
Rhizobium leguminosarum bv. viciae	USDA 2370	T	NR_044774.1	
Rhizobium leguminosarum bv. trifolii	R6-1			AB721425.1
Rhizobium leguminosarum bv. phaseoli	LPA1410			JF792192.1
Rhizobium etli	CFN42	T	NR_074499.1	
Agrobacterium fabrum	C58	T	NR_074266.1	
Rhizobium galegae	LMG6214	T	NR_118990.1	
Agrobacterium vitis	LMG8750	T	NR_118989.1	
Sinorhizobium fredii	NGR234		NR_102919.1	
Sinorhizobium fredii	USDA205	T	NR_112784.1	
Sinorhizobium meliloti	LMG6133	T	NR_118988.1	
Mesorhizobium loti	MAFF303099	T	NR_074162.1	
Bradyrhizobium japonicum	USDA110			L23331.1
Rhodopseudomonas palustris	J			EU531568.1
Bradyrhizobium elkanii	USDA61			AB231916.1
Azorhizobium caulinodans	NBRC14845	T	NR_113675.1	
Aquabacter spiritensis	SPL-1	T	NR_104747.1	
**Strains not described in Figure 4**				
Brucella abortus				X13695
Bartonella bacilliformis				Z11683
Agrobacterium rubi				X67228
Rhizobium aggregatum				X73041
Sinorhizobium saheli				X68390
Sinorhizobium terangae				X68387
Mesorhizobium ciceri				U07934
Mesorhizobium huakuii				D12797
Phyllobacterium myrsinacearum				D12790
Mycoplana dimorpha				D12786
Afipia clevelandensis	ATCC49720			M69186
Afipia felis	ATCC53690			M65248

Name	Strain	Type strain (T)	Source of 16S rRNA sequence	
			NCBI RefSeq	Genbank
Nitrobacter hamburgensis				L11663
Nitrobacter winogradskyi				L11661
Bradyrhizobium denitrificans				S46917
Methylobacterium organophilum				D32226
Beijerinckia indica				M59060
Starkeya novella				D32247
Ancylobacter aquaticus				M62790
Rhodoplanes elegans				D25311
Blastochloris viridis				D25314
Rhodomicrobium vannielii				M34127
Rhodospirillum rubrum				D30778

Supplementary Table 4. Plants and sequences used for phylogeny

Name	Source of *rbcL* sequence (Genbank)
Galega officinalis	KM360795.1
Vicia sativa	NC_027155.1
Pisum sativum	NC_014057.1
Melilotus officinalis	JX848463.1
Trifolium subterraneum	NC_011828.1
Medicago truncatura	NC_003119.6
Lotus japonicus	NC_002694.1
Anthyllis vulneraria	KF602115.1
Sesbania vesicaria	KJ773882.1
Phaseolus vulgaris	NC_009259.1
Vigna unguiculata	NC_018051.1
Glycine max	NC_007942.1
Cajanus cajan	Z95535.1
Lupinus albus	NC_026681.1
Leucaena trichandra	NC_028733.1
Parasponia parviflora	AF500342.1
